# Responses of *Mytilus galloprovincialis* in a Multi-Stressor Scenario: Effects of an Invasive Seaweed Exudate and Microplastic Pollution under Ocean Warming

**DOI:** 10.3390/toxics11110939

**Published:** 2023-11-18

**Authors:** Cristiana Lopes, Andreia C. M. Rodrigues, Sílvia F. S. Pires, Diana Campos, Amadeu M. V. M. Soares, Hugo C. Vieira, Maria D. Bordalo

**Affiliations:** 1 Department of Biology, University of Aveiro, 3810-193 Aveiro, Portugal; cristiana12@ua.pt; 2 CESAM—Centre for Environmental and Marine Studies, Department of Biology, University of Aveiro, 3810-193 Aveiro, Portugal; rodrigues.a@ua.pt (A.C.M.R.); silviapires1@ua.pt (S.F.S.P.); diana.campos@ua.pt (D.C.); asoares@ua.pt (A.M.V.M.S.); hugovieira@ua.pt (H.C.V.)

**Keywords:** invasive species, climate change, microplastics, mussels, oxidative stress-related biomarkers

## Abstract

Microplastic pollution, global warming, and invasive species are known threats to marine biota, but the impact of their simultaneous exposure is still not well understood. This study investigated whether the toxic effects posed by the invasive red seaweed *Asparagopsis armata* exudate (2%) to the mussel *Mytilus galloprovincialis* are amplified by a 96 h exposure to increased temperature (24 °C) and polyethylene microplastics (PE-MPs, 1 mg/L). Biochemical (neurotoxicity, energy metabolism, oxidative stress, and damage) and physiological (byssal thread production) responses were evaluated. The number of produced byssus greatly decreased under concomitant exposure to all stressors. The antioxidant defences were depleted in the gills of mussels exposed to temperature rises and PE-MPs, regardless of exudate exposure, preventing oxidative damage. Moreover, the heat shock protein content tended to decrease in all treatments relative to the control. The increased total glutathione in the mussels’ digestive gland exposed to 24 °C, exudate, and PE-MPs avoided oxidative damage. Neurotoxicity was observed in the same treatment. In contrast, the energy metabolism remained unaltered. In conclusion, depending on the endpoint, simultaneous exposure to *A. armata* exudate, PE-MPs, and warming does not necessarily mean an amplification of their single effects. Studies focusing on the impact of multiple stressors are imperative to better understand the underlying mechanisms of this chronic exposure.

## 1. Introduction

Coastal ecosystems, besides sheltering more than one-third of the world’s human population and providing several services, represent areas of high biodiversity and productivity that are increasingly being threatened by anthropogenic pollution [1,2], biological invasions [3], and stress induced by climate change [4].

In particular, biological invasions are a growing concern, causing adverse economic, social, and ecological impacts [3]. Rapid globalisation, international trade, and the growing trend of maritime travel have increased the presence of invasive species in various parts of the globe [5,6]. When communities of non-native species establish in a given territory, they compete with resident organisms, altering the trophic chains, and may ultimately eliminate native organisms, decreasing the genetic variability and favouring homogenisation [7]. Seaweeds are a significant component of biological invasion in marine ecosystems [8]. Global estimates point to 163 to >300 introduced species of seaweed, with their introduction and spread increasing exponentially in the last 20 years [9].

*Asparagopsis armata* Harvey, 1855 is a red seaweed native to southern Australia and New Zealand that was included in the Portuguese list of invasive species [10] and the list of the 100 most invasive species in the Mediterranean [7,11]. *A. armata* can be found in subtidal areas, but also in upper intertidal rocky shores, where they may be found on the surface during low tide [11,12]. This species has few predators, a great adaptation to physical and chemical changes, and the capacity to spread rapidly, colonising vast areas, and potentially affecting the composition of native communities [11,12]. Moreover, the gametophyte phase of this seaweed has vesicles or glandular cells responsible for storing numerous secondary metabolites, such as halogenated compounds (e.g., halomethanes, haloalkanes, haloacids, and halocetones), which are released via exudation for chemical defence against different organisms [13,14]. 

Climate change, particularly rising temperatures, has also become an increasing concern and threat to coastal ecosystems [15]. Activities such as burning fossil fuels release large amounts of CO_2_ and other greenhouse gases into the atmosphere, causing the oceans and atmosphere to warm above normal levels [16,17]. From 1971 to 2010, the ocean surface is known to have warmed at about 0.11 °C per decade, with a maximum recorded in coastal areas of approximately 0.18 °C [16]. Bivalves are highly susceptible under these scenarios since they are sessile and ectothermic and inhabit subtidal or intertidal zones [17]. Therefore, they are prone to more pronounced and regular temperature fluctuations [18].

Among pollutants, plastic contamination is one of the biggest issues that coastal organisms and ecosystems face [19]. In particular, microplastics (MPs), ubiquitous pollutants in all oceans with a size of < 5 mm, represent about 93% of the plastic waste in the marine environment, being rapidly incorporated by different marine organisms and transported along the food chain [20,21]. Once in the aquatic compartment, MPs can exert toxic effects on multiple marine organisms; however, they must be bioavailable, which will depend on both the pollutant’s properties and the organisms’ ingestion preferences [22]. Bivalves are among the marine organisms most exposed to the ingestion of MPs due to their filtering behaviour [21]. When filtering seawater to feed, they can ingest a set of particles that can cause impacts on growth, reproduction, feeding, and survival, being therefore considered a particularly important group to study the effects of MPs [21].

The mussel *Mytilus galloprovincialis* (Lamarck, 1819), native to the Mediterranean Sea, is a sessile species inhabiting subtidal and intertidal areas [11]. Due to their sedentary lifestyle, wide distribution, abundance, tolerance to various environmental conditions, ease of handling, and filtering behaviour, these organisms are often used as biological indicators of pollution [23,24]. Additionally, toxicity assays with mussels are relatively easy to perform, rapid, and highly cost-effective [24]. *M. galloprovincialis* also play a crucial role in marine trophic chains, so any disturbance to these individuals may lead to drastic effects on the functioning of these ecosystems [8,23]. Furthermore, owing to their byssal thread production, these bivalves have a high capacity to generate large clusters on rocky shores (mussel beds), providing shelter to many species, and thus increasing habitat complexity and contributing to increased biodiversity [11]. They also have a pivotal role in improving water quality by filtering particles and excess nutrients and consequently reducing water turbidity [25]. When thermally stressed, bivalves like *M. galloprovincialis* may show several responses, such as altered cardiac activity that may lead to the closure of valves to avoid additional stress, altered oxygen uptake rates, or a change from aerobic to anaerobic metabolism [26]. In addition to direct impacts, studies show that in a global warming scenario, where the thermal tolerance ranges of marine organisms are exceeded, there may be a change in the sensitivity of organisms to various pollutants with a potential change in their toxicity and bioavailability [18,27,28,29].

Considering that, in the environment, organisms face multiple stressors simultaneously, it is of utmost importance to understand how they respond to these complex scenarios. To the best of our knowledge, a study combining the effects of an invasive seaweed exudate, warming, and microplastic pollution has never been conducted. In this context, this study aimed to evaluate whether the toxic effects posed by *A. armata* exudate to *M. galloprovincialis* are altered when exposed to increased temperature and the presence of MPs. Polyethylene (PE-MPs) was chosen as a model microplastic since it is one of the most common polymers in the marine environment, used for various purposes such as packaging, transportation, and construction, among others [30]. Moreover, due to its low density, PE-MPs can be found in the water column that is bioavailable to marine organisms [31].

## 2. Materials and Methods

### 2.1. Sampling and Acclimation of Mytilus galloprovincialis

In September 2021, specimens of *M. galloprovincialis* were collected by hand near the mouth of the Ria de Aveiro (40°38′38.8″ N 8°44′44.6″ W) during low tide. After sampling, the individuals were immediately transported to the laboratory at the Biology Department, University of Aveiro, and cleaned of epibionts and surface debris. The mussels were then acclimated for 7 days in aquaria with artificial seawater in a recirculation system, allowing continuous water renewal under controlled conditions (salinity 35 ± 0.5; pH 8.0 ± 0.1, O_2_ saturation > 80%), a photoperiod of 14 h light/10 h dark, and a temperature of 20.0 ± 1 °C. Mussels exposed to warming treatments were acclimated to 24.0 ± 1 °C.

### 2.2. Sampling of Asparagopsis armata and Exudate Production

The gametophyte phase of the invasive seaweed *A. armata* was collected in September 2019 whilst snorkelling in the subtidal zone of Angra Bay, Terceira Island, Azores (38°38′59.2″ N 27°13′16.4″ W). After sampling, the seaweeds were kept in tanks with seawater from the site, properly aerated, until they were packed in sealed tanks for transport to the laboratory at the University of Aveiro. After arriving at the laboratory, the seaweeds were cleaned of any organisms and associated debris and allocated for 24 h to a tank with artificial seawater at a ratio of 1:10 (1 kg of seaweed for every 10 L of water) under optimised conditions (salinity 35 ± 1, pH 8.0 ± 0.1, temperature 20.0 ± 0.5 °C), in the dark. After this period, the *A. armata* was removed from the tank, and the resulting medium (stock solution representing 100% of the exudate) was preserved at −20 °C until further use.

### 2.3. Microplastics Solution Preparation

Low-density polyethylene (PE-MPs) powder particles with an irregular shape were purchased from Sigma-Aldrich, UK (CAS: 9002-88-4, size: 34–50 µm, density: 0.94 g/mL at 25 °C). The stock solution (100 mg of PE-MPs/L) was prepared in previously filtered artificial seawater (salinity of 35; salt mixed with reverse osmosis water). Additionally, a solution containing only filtered artificial seawater was also prepared for use in the treatments without added PE-MPs. Both solutions were placed for three days in the dark at room temperature on an orbital shaker at 50 rpm for ageing the MPs used in the assay.

### 2.4. Experimental Setup

After acclimation, 96 mussels (4.6 ± 0.3 cm shell length) were randomly distributed in 1 L glass aquaria containing 500 mL of artificial seawater (1 mussel per aquarium), corresponding to four different treatments at 20 °C and four treatments at 24 °C: (i) the control treatment (pre-filtered artificial seawater only), (ii) *A. armata* exudate (2%), (iii) PE-MPs (1 mg/L) and (iv) simultaneous exposure to *A. armata* exudate (2%) and PE-MPs solution (1 mg/L). No mortality was recorded during the exposure in any of the treatments. The temperatures were kept stable using a system of independent water baths (one per temperature) consisting of a refrigerator (Hailea, HC-300A, Chaozhou, China) and a submersible thermostat (Prodac Magictherm 150 W, Cittadella, Italy).

The temperature of 24 °C was chosen to simulate a scenario of temperature increase predicted for 2100 [32]. The exudate concentration used (2%) was chosen according to the results of sub-lethal toxicity tests performed by Coelho et al. [11]. Each treatment consisted of 12 replicates; from these, seven replicates were used for further biochemical analyses and five replicates for the quantification of MPs. The physicochemical parameters were measured throughout the exposure and were maintained as in the acclimation period. After 96 h, all individuals were inspected for the number of produced byssus (threads connected to the mussel and attached to the glass were counted before removing the organism from the aquarium) and dissected for tissue separation from the gills, digestive gland and muscle. Samples for biochemical analysis were frozen in liquid nitrogen and stored at −80 °C until used. Samples for the MP quantification were frozen in glass vials at −20 °C. In these same five replicates of each treatment, biodeposits (faeces and pseudofaeces) were gently collected with a pipette, stored in microtubes and washed with ultrapure water.

### 2.5. Extraction and Quantification of Microplastics

The extraction and quantification of MPs in *M. galloprovincialis* tissues and biodeposits were performed following a protocol adapted from Campos et al. [33]. The tissues were first weighed and subsequently digested by adding 5 mL of nitric acid (HNO_3_, 65%) to each flask, covered with pierced aluminium foil, and incubated at 60 °C for 3 h. After that, the samples were allowed to cool to room temperature and 4.33 mL of hydrogen peroxide (H_2_O_2_, 35%) was added. The samples were kept overnight at room temperature for subsequent filtration. Then, each sample was diluted in 100 mL of ultrapure water and vacuum filtered using a sterile, gridded cellulose nitrate membrane filter with a 0.45 µm pore size (PRATDUMAS, Couze-St-Front, France). Between treatments, the filtration system was washed with acid and ultrapure water. For quality control, procedural blanks (1 every ten samples) were prepared following the same procedure as the other samples. After drying at room temperature in Petri dishes Ø 55 mm, the filters were observed using a 0.65x–5.0x range magnifier (Zeiss Stemi 2000-C, Feasterville-Trevose, PA, USA) in order to quantify the PE-MPs. In case of doubt about the presence or absence of MPs, the needle technique was used [34]. The number of PE-MPs is presented as the number of particles/g of tissue/organism.

Regarding the biodeposits, the samples previously preserved at −20 °C were allowed to thaw and centrifuged at 1500× *g* at a temperature of 20 °C for 5 min, adapted from Evan et al. [35]. After the first centrifugation, the excess water was removed and 1 mL of ultrapure water was added. Then, the samples were centrifuged again under the same conditions described above and the excess water was removed. The process was repeated three times to eliminate the PE-MPs present in the aqueous medium. Subsequently, the extraction and quantification of MPs were performed as explained above.

### 2.6. Biochemical Analysis

#### 2.6.1. Sample Preparation

The previously dissected tissues (gills, digestive gland and part of the muscle tissue used for oxidative stress analysis) were individually homogenised on ice using a sonicator (10% pulsed mode, 250 Sonifier, Branson Ultrasonics, Danbury, CT, USA) after the addition of 1600 µL of 0.1 M K-phosphate buffer, pH 7.4. Muscle samples to be analysed for energy metabolism were homogenised using the same procedure, only this time in 1600 µL of ultrapure water instead of the buffer.

After homogenisation, an aliquot of each replicate of the three tissues was stored with 8 µL of 4% butylated hydroxytoluene (BHT) in methanol to evaluate the lipid peroxidation (LPO). An aliquot for protein carbonylation (PC) determination and one for heat shock proteins were also taken directly from the homogenate. The remaining homogenate from the gills and digestive gland was centrifuged for 15 min at 10,000× *g* (4 °C). The resulting supernatant was split for a subsequent analysis of catalase (CAT), glutathione-*S*-transferase (GST) and acetylcholinesterase (AChE) activities, as well as the total glutathione content (tGSH). Prior to the enzymatic activity analysis, the microtubules were kept at −80 °C. The resulting product from the homogenisation of the muscle tissue was used to determine the AChE activity.

The aliquots of the homogenate reserved for the energy metabolism analyses were stored at −80 °C for a further analysis of lactate dehydrogenase (LDH), electron transport system (ETS) and protein, lipid, and sugar content.

All biomarker determinations were performed in microassays in 96-well flat-bottom plates and read spectrophotometrically with the MultiSkan Spectrum microplate reader (Thermo Fisher Scientific, Waltham, MA, USA).

#### 2.6.2. Neurophysiological and Oxidative Stress Biomarkers

The protein concentration of PMS was determined according to the Bradford method [36], using bovine γ-globulin as the standard. The AChE activity was measured using acetylthiocholine as a substrate and following the increase in absorbance at 412 nm, as described by the Ellman method [37], adapted for microplates [38,39]. The catalase (CAT) activity was measured from the PMS following the decomposition of the H_2_O_2_ substrate at 240 nm [40]. The glutathione-S-transferase (GST) activity was determined in PMS from the conjugation of GSH with 1-chloro-2,4-dinitrobenzene (CDNB) at 340 nm [41]. Total glutathione (tGSH) content was determined in PMS at 412 nm via a recycling reaction of reduced glutathione (GSH) with 5,5′-dithiobis-(2-nitrobenzoic acid) (DTNB) in the presence of excess glutathione reductase (GR) [42,43]. The determination of lipid peroxidation (LPO) was measured with thiobarbituric acid reactive substances (TBARSs) at 535 nm [44]. The protein carbonylation (PC) was measured based on the reaction of carbonyl groups with 2,4-dinitrophenylhydrazine (DNPH) at 450 nm according to the alkaline DNPH method [45]. The determination of the content of HSP70/HSC70 heat shock proteins was analysed via an ELISA and performed as described by Vieira et al. [46] by sequentially adding a primary antibody (1° Anti-HSP70 mouse mAB (C92F3A-5), Millipore, used for the detection of the 72 and 73 kDa proteins that correspond to the molecular mass of the inducible hsp and hsc70) and a secondary antibody (2° Anti-mouse IgC (fab specific), Sigma). The absorbance was read at 405 nm and a purified HSP70 active protein (HSP70, Millipore) was used as the standard.

#### 2.6.3. Biomarkers of Energy Metabolism

The total lipid content was determined by adding chloroform, methanol and ultrapure water in a ratio of 2:2:1 to 300 μL of the homogenate. After centrifugation, sulfuric acid (H_2_S0_4_) was added to the organic phase of each sample and, right after, incubated at 200 °C for 15 min. The absorbance was measured at 375 nm using tripalmitin as the lipid standard.

To a second homogenate aliquot (300 µL), 15% TCA was added and incubated for 10 min at −20 °C. After centrifugation (1000× *g* for 10 min at 4 °C), carbohydrate quantification was performed on the supernatant by adding 5% phenol and H_2_SO_4_ to the samples, with glucose as the standard, at 492 nm. The pellet was resuspended with 1 M NaOH (incubated for 30 min at 60 °C) and then neutralised with 1.67 M HCl. The Bradford method was used to quantify the total protein content at 520 nm; the standard used was bovine serum albumin [36]. The available energy fractions were converted into equivalent energy values using the corresponding combustion energy: 39,500 mJ/g lipid, 17,500 mJ/g glycogen, 24,000 mJ/g protein [47].

The ETS activity was evaluated using the INT (Iodonitrotetrazolium) reduction assay. The measure of INT’s reduction rate at 490 nm was made possible through the presence of Triton X-100, a non-ionic detergent. To calculate the rate of cellular oxygen consumption, a stoichiometric relationship was used, where, for each 2 μmol of formazan formed, 1 μmol of oxygen is consumed. To convert the final Ec value into energy equivalents, the specific enthalpic equivalent for an average mixture of lipids, proteins and carbohydrates of 480 kJ/mol O_2_ was used [47].

The samples used for the LDH determination were subjected to freezing/melting and then centrifuged at 1000× *g* for 10 min at 4 °C. The supernatant resulting from the centrifugation was used to determine the LDH activity by decreasing the absorbance at 340 nm, since, with the consumption of pyruvate, there would be a consequent oxidation of NADH [48] adapted for microplates [11].

### 2.7. Statistical Analysis

Statistical analyses of the data and graph presentation were performed using GraphPad Prism v.9 (GraphPad Software, La Jolla, CA, USA). The normality of residuals was checked using a Kolmogorov–Smirnov test and equal variances were checked using Spearman’s test for heteroscedasticity. A 3-way ANOVA was performed using the exudate concentration, the PE-MP concentration and the temperature as fixed factors. Variables that did not show normal distribution or homoscedasticity were log_10_ transformed (CAT, GST, tGSH, AChE, LPO and PC in the gills, CAT AChE and HSP70 in the digestive gland and AChE, LPO, PC, protein and lipid content in the muscle). Multiple comparisons were performed using Sidak’s test to find significant differences between treatments. Significance was accepted at *p* < 0.05. The graphs are presented with the mean value (mean) ± the standard error of the mean value (SEM).

## 3. Results

### 3.1. Quantification of PE-MPs in M. galloprovincialis

PE microparticles were found mostly in the digestive gland and less in the gills (Table 1). In each temperature scenario, a tendency for an increased number of particles was observed in the digestive gland of mussels exposed to *A. armata* exudate. On the other hand, in each exudate concentration scenario, a lower accumulation of PE-MPs was also observed in the digestive gland in the 24 °C treatments when compared to 20 °C (Table 1). However, these differences were not significant (*p* > 0.05). Regarding the gills, there were no significant differences between treatments nor any clear tendency (*p* > 0.05, Table 1). In the biodeposits, despite the observed increase in the number of PE-MPs in mussels exposed to 20 °C when compared to the ones at 24 °C (Table 1), this difference was not significant (*p* > 0.05). Also, no differences were found regarding the presence of *A. armata* exudate (*p* > 0.05).

### 3.2. Biomarkers

#### 3.2.1. Neurophysiological and Oxidative Stress Biomarkers

**Figure 1:**
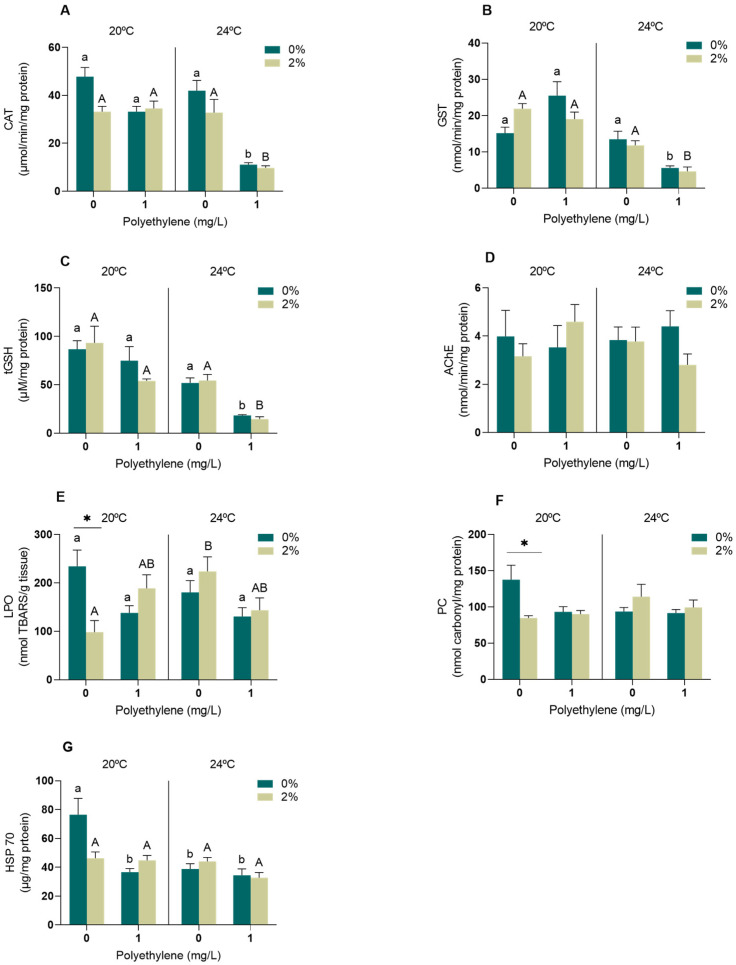
Oxidative stress biomarkers in the gills of *M. galloprovincialis* exposed for 96 h to *A. armata* exudate (0 and 2%) to different concentrations of PE-MPs (0 and 1 mg/L), at two different temperatures (20 and 24 °C). (**A**) catalase activity (CAT), (**B**) glutathione-S-transferase activity (GST), (**C**) total glutathione content (tGSH), (**D**) acetylcholinesterase activity (AChE), (**E**) lipid peroxidation (LPO) (**F**) protein carbonylation (PC) and (**G**) heat shock proteins (HSP70). All values represented in the graphs are presented as mean ± SEM. * indicates a significant difference between the 0 and 2% treatments of *A. armata* exudate for the same concentration of PE-MPs. Lower case letters represent differences between the 0% exudate treatments, while upper case letters indicate differences between the 2% exudate treatments.

In the gills, significant effects were observed on the CAT activity as a result of the interaction between the temperature and PE-MPs (*p* < 0.05, Table S1). The CAT activity reduced the most in organisms that were simultaneously exposed to PE-MPs at 24 °C in both exudate concentrations (Figure 1A). Regarding GST, significant effects were observed regarding the interactions of PE-MPs vs. temperature and PE-MPs vs. exudate (*p* < 0.05, Table S1). GST showed a similar trend to CAT, and a reduction in its activity was observed, presenting significant differences upon exposure to PE-MPs at 24 °C, within each of the 0 and 2% exudate concentrations, when compared to any of the other treatments (Figure 1B). Significant alterations were also observed in the tGSH levels due to the PE-MP and temperature interaction (*p* < 0.05, Table S1). There was a decrease in tGSH in the gills of mussels exposed to PE-MPs at 24 °C at both the 0% and 2% exudate concentrations (Figure 1C). Regarding the AChE activity (Figure 1D), no significant differences were observed in the mussels exposed to any of the treatments (*p* > 0.05, Table S1). On the other hand, there was a significant interaction in the LPO levels among PE-MPs, temperature and exudate (*p* < 0.05, Table S1). LPO significantly increased in mussels exposed to 2% exudate and an increased temperature of 20 °C (Figure 1E), and a reduction was observed between the 0% and 2% concentrations of exudate at 20 °C in the absence of PE-MPs (Figure 1E). Finally, an interaction between temperature and *A. armata* exudate was found to significantly affect the PC levels (*p* < 0.05, Table S1). A significant decrease was observed in the mussels exposed to 2% exudate relative to 0% at 20 °C in the absence of PE-MPs (Figure 1F). Regarding HSPs, a significant interaction was observed between the temperature, exudate and PE-MPs (*p* < 0.05, Table S1). The mussels exposed to the control conditions (20 °C, 0% exudate and no PE-MPs) presented the highest HSP levels in relation to the other treatments (Figure 1G).

**Figure 2:**
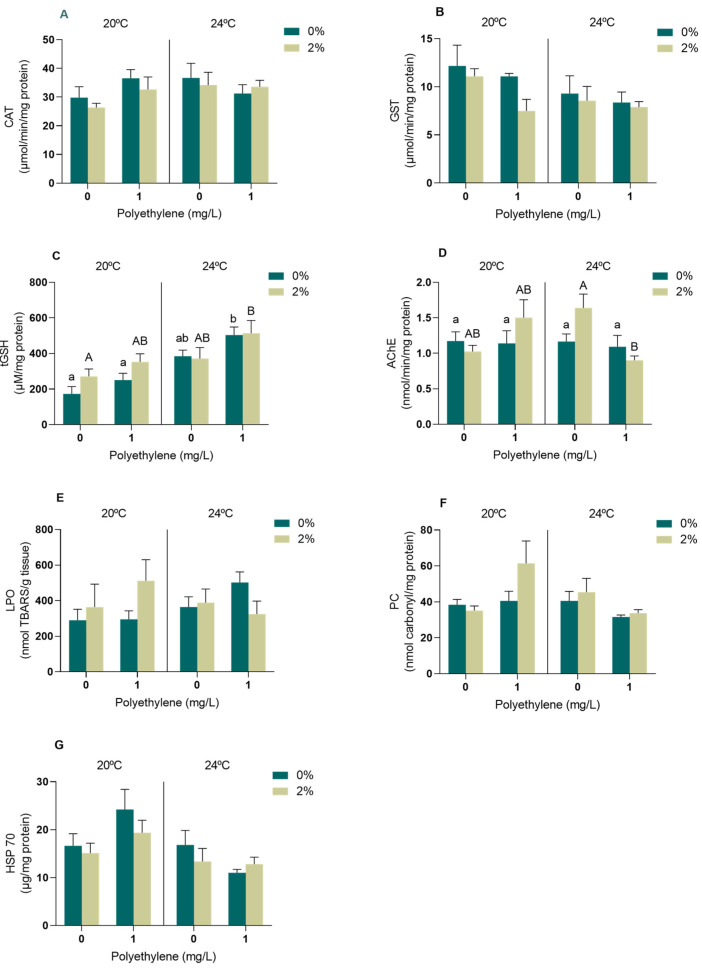
Oxidative stress biomarkers in the digestive gland of *M. galloprovincialis* exposed for 96 h to *A. armata* exudate (0 and 2%) to different concentrations of PE-MPs (0 and 1 mg/L) at two different temperatures (20 and 24 °C). (**A**) Catalase activity (CAT), (**B**) glutathione-S-transferase activity (GST), (**C**) total glutathione content (tGSH), (**D**) acetylcholinesterase activity (AChE), (**E**) lipid peroxidation (LPO), (**F**) protein carbonylation (PC) and (**G**) heat shock proteins (HSP70). All values represented in the graphs are presented as mean ± SEM. Lower case letters represent differences between the 0% exudate treatments, while upper case letters indicate differences between the 2% exudate treatments.

In the digestive gland, no significant differences (*p* > 0.05) were observed in CAT activity in any of the treatments (Table S2, Figure 2A), while significant effects were detected in the GST activity regarding temperature (*p* < 0.05, Table S2); however, these differences were not identified by post hoc tests (Figure 2B). Regarding tGSH content, significant effects were observed in the digestive gland of mussels exposed to PE-MPs and temperature (*p* < 0.05, Table S2).

Increased tGSH levels were identified in the digestive glands of the mussels exposed to the *A. armata* exudate, PE-MPs and 24 °C when compared to those exposed to the exudate at 20 °C without PE-MPs (Figure 2C). Significant differences in tGSH levels were also found in the mussels exposed to no exudate, increasing in the 24 °C treatment with PE-MPs when compared to both 20 °C treatments (with and without PE-MPs, Figure 2C). Concerning the AChE activity in the digestive gland of *M. galloprovincialis*, significant effects were identified in the interactions among the exudate, PE-MPs and temperature (*p* < 0.05, Table S2), decreasing in mussels that were exposed to the exudate and 24 °C in the presence of PE-MPs (Figure 2D). Regarding oxidative damage, no significant alterations were observed at the lipid (*p* > 0.05, Table S2, Figure 2E) and protein (*p* > 0.05, Table S2, Figure 2F) levels in mussels exposed to any of the treatments. On the other hand, an interaction between PE-MPs and temperature was detected in the HSP70 heat shock proteins of the digestive gland of exposed mussels (*p* < 0.05, Table S2); however, significant differences between treatments were not identified in the post hoc tests (Figure 2G).

**Figure 3:**
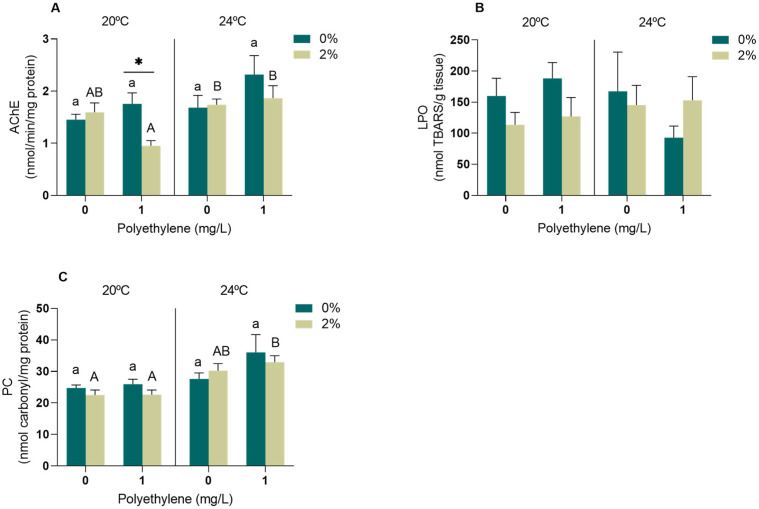
Oxidative stress biomarkers in the muscle of *M. galloprovincialis* exposed for 96 h to *A. armata* exudate (0 and 2%) and different concentrations of PE-MPs (0 and 1 mg/L) at two different temperatures (20 and 24 °C). (**A**) Acetylcholinesterase activity (AChE), (**B**) lipid peroxidation (LPO), (**C**) protein carbonylation (PC). All values represented in the graphs are presented as mean ± SEM. * Indicates a significant difference between the 0 and 2% treatments of *A. armata* exudate for the same concentration of PE-MPs. Lower case letters represent differences between the 0% exudate treatments, while upper case letters indicate differences between the 2% exudate treatments.

In the muscle, significant effects on the AChE activity (*p* < 0.05) were attributed to the interactions of PE-MPs vs. temperature and PE-MPs vs. exudate (Table S3). In particular, the AChE activity in mussels exposed to PE-MPs at 20 °C decreased in the presence of the exudate. In addition, the AChE activity increased in mussels exposed to PE-MPs and exudate at 20 °C compared to those exposed to the exudate at 24 °C, both with and without the presence of PE-MPs (Figure 3A). LPO did not experience significant alterations (*p* > 0.05) in mussels exposed to any of the studied factors or their interaction (Table S3, Figure 3B). Regarding the PC levels, significant effects were detected for temperature (*p* < 0.05) (Table S3). Oxidative damage at the protein level increased in the mussels exposed to 2% exudate and PE-MPs at 24 °C when compared to mussels exposed to the exudate at 20 °C in both PE-MP treatments (Figure 3C).

#### 3.2.2. Biomarkers of Energy Metabolism

**Figure 4:**
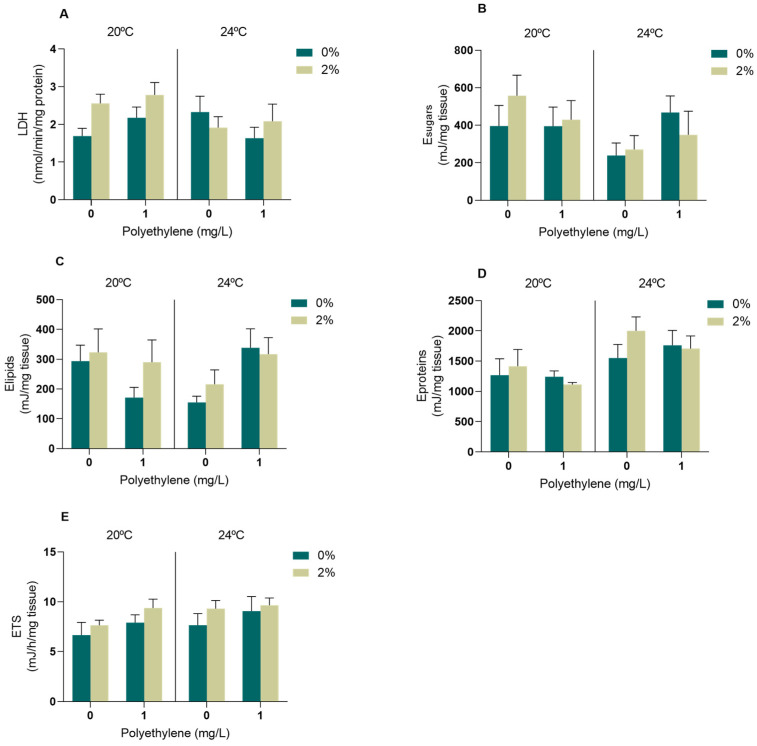
Energy metabolism biomarkers in the muscle of *M. galloprovincialis* exposed for 96 h to *A. armata* exudate (0 and 2%) and different concentrations of PE-MPs (0 and 1 mg/L) at two different temperatures (20 and 24 °C). (**A**) Lactate dehydrogenase activity (LDH), (**B**) sugar content (E sugars), (**C**) lipid content (E lipids), (**D**) protein content (E protein) and (**E**) electron transport system activity (ETS). All values represented in the graphs are presented as mean ± SEM.

Considering the energy metabolism evaluated in the muscle tissue, the LDH activity (Figure 4A), sugar content (Figure 4C) and aerobic energy production measured as ETS activity (Figure 4E) were not altered in the presence of the *A. armata* exudate, PE-MPs or increased temperature, and there was no interaction between any of the factors (*p* > 0.05, Table S4). On the other hand, the interaction between PE-MPs and temperature resulted in a significant effect on the lipid content (Table S4); however, no significant differences were identified between treatments (Figure 3C). Regarding the protein content, a significant effect of temperature was observed, although post hoc tests did not identify statistical differences between the treatments (Table S4, Figure 4D).

### 3.3. Byssal Thread Production in M. galloprovincialis

**Figure 5:**
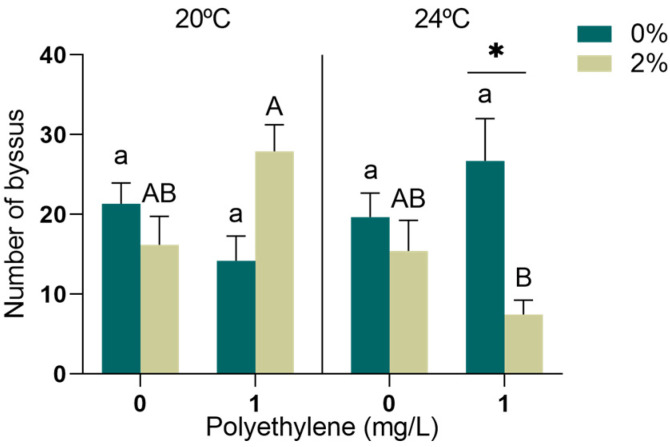
Number of byssus produced by *M. galloprovincialis* exposed for 96 h to *A. armata* exudate (0 and 2%), for different concentrations of PE-MPs (0 and 1 mg/L) at two different temperatures (20 and 24 °C). All values represented in the graphs are presented as mean ± SEM. * Indicates a significant difference between the 0 and 2% treatments of *A. armata* exudate for the same concentration of PE-MPs. Lower case letters represent differences between the 0% exudate treatments, while upper case letters indicate differences between the 2% exudate treatments.

The interaction of the three factors significantly altered the number of functional byssus produced by *M. galloprovincialis* (*p* < 0.05, Table S5). The number of produced byssus was significantly reduced in mussels exposed to 2% exudate and 1 mg/L of PE-MPs simultaneously at 24 °C when compared to the same treatment at 20 °C (Figure 5). A significant decrease was also detected in the mussels exposed to 2% exudate compared to 0% when simultaneously exposed to 1 mg/L of PE-MPs at 24 °C (Figure 5).

## 4. Discussion

### 4.1. PE-MPs in M. galloprovincialis Tissues and Biodeposits

PE-MPs were mostly found in the digestive gland of *M. galloprovincialis,* indicating that these particles were captured, ingested and transferred to the digestive gland to undergo digestion [23,49,50,51]. On the other hand, due to the size of the PE-MPs (34–50 µm) used in this study, an internalisation of the particles in the gill tissues is not expected [52], which means that the small number of particles found in the gills have probably adhered to the gill epithelium. In both temperature scenarios, PE-MPs accumulated the most in the digestive gland of the mussels exposed to the *A. armata* exudate, as reported by Rodrigues et al. [23] after exposing *M. galloprovincialis* to polyamide microplastics (PA-MPs). These authors hypothesised that the metabolites present in the exudate could have compromised the excretion capacity of the mussels. However, using the number of PE-MPs in the biodeposits as a proxy for the particles’ excretion, no observable differences were found in the presence or absence of *A. armata* exudate. Thus, further studies are needed to understand the mechanisms underlying this pattern. In contrast, a reduced particle accumulation in the digestive gland occurred mainly when organisms were exposed to 24 °C, which may be explained by the extended periods of valve closure that are expected at this temperature as a protective mechanism [52]. With the likely closure of the valves, mussels tend to ingest fewer particles, resulting in less accumulated PE-MPs in the digestive gland and less excreted particles in the biodeposits [49]. However, behavioural responses regarding clearance rates and valve opening/closing were not evaluated in this study.

### 4.2. Biomarkers

#### 4.2.1. Neurophysiological and Oxidative Stress Biomarkers

The toxicity induced by the MPs, temperature and exudate is largely mediated by excess of reactive oxygen species (ROS), leading to the action of the antioxidant defences to maintain the redox balance and avoid oxidative damage [20]. In bivalves such as *M. galloprovincialis*, the gills are the first organ exposed to different types of stressors; thus, respiratory and feeding functions can be affected when in contact with them [53]. In the gills, the activity of CAT, an enzyme that acts as a first line of defence against oxidative stress, was significantly reduced in organisms exposed to increased temperature and PE-MPs regardless of exudate concentrations. The excessive production of ROS favours the formation of H_2_O_2_ (hydrogen peroxide), which is reduced by CAT to H_2_O and O_2_. Thus, a decline in CAT activity indicates a strong involvement of this enzyme in the neutralisation of excess ROS. These results agree with Rodrigues et al. [23], who also observed a tendency for reduction in CAT activity in the gills of *M. galloprovincialis* under exposure to PA-MPs. Other studies that exposed *M. galloprovincialis* to different temperature scenarios (20 vs. 24 °C and 17 vs. 22 °C) also demonstrated a tendency for decreased CAT activity with increasing temperature [8,54]. GST, a phase II biotransformation enzyme that aims to increase the hydrophilicity of the compounds to favour its excretion [55], also decreased activity in organisms exposed to warming and PE-MPs, both in the presence and absence of the exudate. A decrease in GST activity with increasing water temperature was also observed by Andrade et al. [56] in *M. galloprovincialis* exposed to 22 °C for 28 days when compared to organisms exposed to 17 °C; however, in contrast to our study, this effect was not maintained when warming was combined with a chemical stressor (the rare-earth element lanthanum). Regarding non-enzymatic antioxidant defences, tGSH levels presented a pronounced decrease in mussels under warming conditions and simultaneously exposed to PE-MPs (with or without the presence of exudate). This decrease might be related to the consumption of total glutathione to neutralise ROS and protect the cell from oxidative damage [57], and was previously observed in the gills of *M. galloprovincialis* exposed simultaneously to PA-MPs and *A. armata* exudate [23]. Although there was a reduction in CAT and GST activities and tGSH levels in organisms exposed simultaneously to increased temperature and PE-MPs, regardless of the presence of exudate, no oxidative damage at the lipid (LPO) and protein (PC) level was observed, indicating that the organisms were able to cope with excess ROS within the 96 h of exposure to these stressors. On the other hand, the reduced LPO and PC in the gills of the mussels exposed to 2% exudate (at 20 °C, no PE-MPs) may be explained by the potent antioxidant activity that is described for several bioactive compounds present in the exudate [58,59]. However, in the case of LPO, when simultaneously exposed to warming, this effect is counteracted by the increased temperature, which is known to be a strong stressor to aquatic animals and, therefore, seems to reveal an extra challenge posed by the combination of both stressors in terms of the oxidative stress status of exposed animals. Regarding neurotoxicity, no effect was observed in the gills under any stress conditions. On the other hand, HSP70 tended to reduce in all treatments when compared to the control, suggesting its active contribution to repairing damaged proteins, therefore avoiding significant damage at the protein level under these scenarios, which may indicate a more toxic response than its up-regulation [60]. HPS70 is a highly conserved protein that is synthesised in order to protect cells against different stressors, being responsible for protein (re)folding, assembly, translocation and degradation [61]. Thus, HSP inhibition may hamper their role as molecular chaperones and in the maintenance of endogenous protein homeostasis. The down- or up-regulation of HSPs depends on the stressor, dose/intensity and duration of exposure [62]. Short-term exposures to elevated temperatures usually result in their up-regulation; however, in our case, we did not observe the translation of this into higher levels of HSPs available after 96 h exposure, possibly due to the previous acclimation of mussels to 24 °C.

In the digestive gland, no alterations in enzymatic antioxidant defences were observed besides the GST activity decreasing with increasing temperature. The CAT activity was also unchanged in the digestive gland of the *M. galloprovincialis* males exposed to concentrations ranging from 1 to 1000 µg/L of PE-MPs [63] and in *M. galloprovincialis* exposed to 20 and 24 °C [64]. A previous study exposing *M. galloprovincialis* simultaneously to 2% exudate and a temperature increase (20 to 24 °C) also did not find alterations between treatments regarding CAT or GST activity in the digestive gland [8]. On the other hand, increased tGSH levels suggest an up-regulation of non-enzymatic mechanisms in the digestive gland as a response to excess ROS production, especially under PE-MP and warming conditions. A study conducted with the fish *Pomatoschistus microps*, exposed to different temperatures (15, 20 and 25 °C), also observed an increase in tGSH levels in the liver for the highest temperature tested [46]. The lower pressure in terms of oxidative stress caused by the studied stressors on the digestive gland is supported by the lack of oxidative damage identified in this organ (LPO and PC). Concerning the AChE activity in the digestive gland, a significant reduction was observed in the organisms exposed to the exudate at 24 °C in the presence of PE-MPs. With AChE inhibition, the hydrolysis of Ach is no longer possible, which leads to an accumulation of the neurotransmitter in the synaptic cleft that may result in disturbances at the neurological level [65]. The inhibition of AChE was also demonstrated in the digestive gland of the clam *Donax trunculus* after being exposed to a mixture of polyethylene (PE) and polypropylene (PP) microparticles [66]. The mechanisms of action of MPs on the neurological system of different marine organisms are currently not well elucidated; however, AChE inhibition with exposure to MPs has been reported in several studies, which should be a factor to consider when evaluating the effects of these particles [50,66,67]. Neurotoxicity has also been reported in different species under temperature rises [64,68,69]. In contrast, exposure to the tested conditions did not alter HSP70 levels in the digestive gland, reinforcing that the gills may be the most suitable organ to study this stress response [70,71].

LPO, PC and AChE were also evaluated in the muscle of *M. galloprovincialis*. The muscle is not considered a major organ of detoxification, but it is important in controlling valve movements that are essential for organism survival [72,73]. Despite the absence of lipid peroxidation, increased PC values indicate oxidative damage at the protein level in the muscle of individuals under warming conditions when exposed simultaneously to the seaweed exudate and PE-MPs. A similar outcome was observed by Vieira et al. [8] after exposing *M. galloprovincialis* simultaneously to 24 °C and 2% *A. armata* exudate. The increased temperature also led to an induction of AChE activity (when simultaneously exposed to 2% exudate), which may represent a regulatory overcompensation under warming conditions. An AChE induction is also expected as a result of inflammatory processes [74] and may be associated with cell rupture or apoptosis [75]. In contrast, under the control temperature and MP exposure, an inhibition of AChE was triggered by the presence of the exudate. *A. armata* is rich in numerous metabolites, including halogenated compounds that have already been described as important enzyme inhibitors [59,76], and may have contributed to AChE inhibition. Furthermore, the possibility of neurotoxic effects may indicate behavioural alterations and corroborate the likelihood of valve closure due to the concomitant exposure to these stressors.

#### 4.2.2. Energy Metabolism Biomarkers

The energy reserves were measured considering the content of lipids, sugars and proteins. These indices are used to evaluate the maintenance of the basal state of organisms to ensure their vital functions and survival and understand the adaptive potential to stress situations [77]. The lipid content was changed due to the interaction of the microplastics’ presence with the temperature. Lipids are highly energetic molecules that play a crucial role in maintaining homeostasis and enabling marine bivalves to withstand periods of starvation, essential for the growth and overall health of mussels [78]. The lack of significant changes in the other two energy reserves evaluated is in accordance with the previous indications of low to moderate oxidative stress imposed by the studied stressors in a short period to *M. galloprovincialis*, as already observed in mussels exposed to *A. armata* exudate and water warming [8]. Nevertheless, there is a tendency for an increase in protein levels with temperature rise, which may be related to the production of proteins involved in detoxification processes and defence mechanisms [79,80]. In a study that exposed *M. galloprovincialis* to 21 °C, it was also possible to see a higher protein content when compared to mussels at 17 °C [80]. Concerning LDH, the activity of this glycolytic enzyme was not altered, suggesting that there was no need for energy mobilisation via the anaerobic pathway to cope with the stress caused by warming, PE-MPs and/or *A. armata* exudate [81]. Energy consumption, measured as mitochondrial electron transport system (ETS) activity, also remained unaltered between the treatments, indicating that in response to these levels of stress, the exposed mussels were able to maintain their metabolic capacity similar to the control values. Thus, the tested conditions and exposure period seemed to be insufficient to generate a change in aerobic metabolism. Similar results were previously observed in other studies testing the exposure of *M. galloprovincialis* to different temperatures (17 and 21 °C) and other contaminants, such as mercury [28], cyclophosphamide [82] and neodymium [83], and also to the seaweed exudate under warming conditions [8]. Energy metabolism was maintained under these scenarios, which is crucial for securing the homeostasis of organisms, ensuring their vital functions and survival [77].

### 4.3. Byssal Thread Production

*M. galloprovincialis* is a sessile organism depending on attachment to the substrate or to other organisms to cope with the hydrodynamic force of waves and tides, and also predation [84,85]. Byssal threads are extracellular collagen structures, secreted by the foot, consisting of adhesive plates that act as a “glue”, allowing the organisms to present effective mechanisms to strongly attach themselves to the substrates [86,87]. If the production of these defensive structures is impaired, individuals may be easily swept away by waves, decreasing their physiological fitness and even survival [86]. Consequently, the preservation of the mussel beds and their function in the ecosystem will be affected, thus decreasing the habitat complexity provided by them and impairing the associated biodiversity [53]. Byssus production constitutes a substantial energetic cost [88]; thus, when under stressful conditions, mussels need to balance their energy between physical and other defences, constraining some normal physiological processes when exposed simultaneously to multiple stressors such as the exudate, PE-MPs and increased temperature. Since the overall energy budget was unaffected, the available energy could be diverted mostly to oxidative stress coping mechanisms, with less energy being allocated to the secretion of new threads. Temperature is critical to byssogenesis [89], and the adverse effects of ocean warming are expected to impact this process [89]. For instance, a dramatic decline in the quality and number of threads has been found previously in the mussel *Mytilus trossulus* after a temperature increase from 10 to 25 °C [90] and in *Mytilus coruscus* from 21 °C and 27 to 31 °C [91]. Furthermore, a decline in byssus production was previously identified in *M. galloprovincialis* exposed to *A. armata* exudate (1 and 2%), which also hampered their byssal attachment strength [11]; however, the chemical mechanisms adjacent to this are still unknown. *M. galloprovincialis* exposed to PA-MPs and simultaneously to 2% *A. armata* exudate have also been shown to reduce their number of byssus [23]. A reduction in the production of byssal threads in two other mytilid species, *M. coruscus* [92] and *Mytilus edulis* [93], was also found in response to MP exposure. A similar outcome was also reported in the mussel *Perna canaliculus,* exposed to PE-MPs [94]. Considering the increasing frequency of these disturbances and the effect observed in this study, it is reasonable to expect that their co-occurrence may result in mussel displacement, predator susceptibility and decreased fertilization success, with a cascading impact on the population density and stability of mussel beds, consequently affecting intertidal communities.

## 5. Conclusions

There is a growing awareness about the possible impacts of microplastic pollution, global warming and invasive species’ presence on native marine organisms; however, their combined effects are still poorly studied. Thus, this study fills this gap, providing relevant information about the effects caused by these three stressors, using as a model organism the bivalve *Mytilus galloprovincialis*, a species with great ecological and economic relevance. These findings showed that a 96 h exposure to different combinations of PE-MPs, increased temperature and *A. armata* exudate (2%) might represent a risk to *M. galloprovincialis*. Specifically, antioxidant defences were triggered, which, although effective in preventing oxidative damage at the lipid level, were not sufficient to avoid protein damage in the muscle. Neurotoxicity has also been identified in the digestive gland after exposure to these stressors. Further, a higher susceptibility of gills to oxidative stress under the tested conditions was observed, which, in the long term, might compromise the animals’ respiratory and feeding behaviour and, ultimately, their survival. Despite the lack of significant alterations in the energy budget of mussels, this short-term exposure may have compromised the energy available for development. This trade-off may explain the reduction in byssus production, compromising the attachment capacity of *M. galloprovincialis* and, consequently, the various functions it performs in coastal areas. Based on the results obtained here, we may conclude that the concomitant exposure to warming and microplastic pollution may interact with *A. armata* exudate toxicity. However, given that the organisms’ responses depend on multiple biochemical and physiological mechanisms, these effects are never straightforward, with different endpoints responding differently to distinct scenarios. Considering that organisms are seldom exposed to one stressor at a time, studies focused on the impact of multiple stressors should be encouraged.

## Figures and Tables

**Table 1:** Number of polyethylene microplastics (PE-MPs) in the soft tissues (digestive gland and gills) and biodeposits of *Mytilus galloprovincialis* exposed to 1 mg/L of PE-MPs and 1 mg/L with 2% *A. armata* exudate, at two temperature scenarios (20 and 24 °C). All values are presented as mean ± SEM.

Treatments	Number of Particles per Tissue Gram (Wet Weight)		Number of Particles
	Digestive Gland	Gills	Biodeposits
PE-MPs (20 °C)	137.5 ± 34.9	30.0 ± 18.3	2605 ± 252.1
PE-MPs + Exudate (20 °C)	272.4 ± 115.7	3.8 ± 0.50	2948 ± 692.7
PE-MPs (24 °C)	50.6 ± 25.5	25.3 ± 11.6	1826 ± 285.9
PE-MPs + Exudate (24 °C)	117.7 ± 25.8	21.5 ± 6.4	1758 ± 304.3

## Data Availability

The data presented in this study are available in the current manuscript; raw data are available on request from the corresponding author.

## Supplementary Materials

The following supporting information can be downloaded at https://www.mdpi.com/article/10.3390/toxics11110939/s1: Table S1: Three-way ANOVA analysis results on biochemical biomarkers responses in the gills of *Mytilus galloprovincialis* with *Asparagopsis armata* exudate, polyethylene microplastics (PE-MPs) and temperature exposures as factors. Table S2: Three-way ANOVA analysis results on biochemical biomarkers responses in the digestive gland of *M. galloprovincialis* with *A. armata* exudate exposure, PE-MPs and temperature exposures as factors., Table S3: Three-way ANOVA analysis results on biochemical biomarkers responses in the muscle of *M. galloprovincialis* with *A. armata* exudate exposure, PE-MPs and temperature exposures as factors. Table S4: Three-way ANOVA analysis results on energy metabolism biomarkers responses in the muscle of *M. galloprovincialis* with *A. armata* exudate exposure, PE-MPs and temperature exposures as factors. Table S5: Three-way ANOVA analysis results on the byssus production of *Mytilus galloprovincialis* with *Asparagopsis armata* exudate, polyethylene microplastics (PE-MPs) and temperature exposures as factors.
