# Supplementary material for: Responses of Mytilus galloprovincialis in a Multi-Stressor Scenario: Effects of an Invasive Seaweed Exudate and Microplastic Pollution under Ocean Warming
Source: Toxics. 2023 Nov 18;11(11):939. doi: 10.3390/toxics11110939 (PMC10675577; doi:10.3390/toxics11110939)
Supplement: Supplementary file 1 [file toxics-11-00939-s001.zip › toxics-2711068-supplementary.pdf]

Table S1. Results of a 3-way ANOVA for the biochemical biomarkers responses in the gills of *M. galloprovincialis* with exposure to *A. armata* exudate, PE-MPs and two different temperatures as factors.

Biomarkers	Factor	df	F	*p*	% Variation
CAT	PE-MPs	1	94.28	**<0.0001**	32.33
	Temperature	1	82.58	**<0.0001**	28.31
	Exudate	1	7.231	**0.0098**	2.479
	PE-MPs x Temperature	1	54.82	**<0.0001**	18.80
	PE-MPs x Exudate	1	4.024	0.505	1.380
	Temperature x Exudate	1	0.1470	0.7031	0.05042
	PE-MPs x Temperature x Exudate	1	0.5671	0.4551	0.1944
GST	PE-MPs	1	14.81	**0.004**	7.769
	Temperature	1	89.48	**<0.001**	46.93
	Exudate	1	0.4875	0.4884	0.2557
	PE-MPs x Temperature	1	30.13	**<0.0001**	15.80
	PE-MPs x Exudate	1	4.956	**0.0307**	2.599
	Temperature x Exudate	1	1.896	0.1749	0.9944
	PE-MPs x Temperature x Exudate	1	0.9063	0.3459	0.4753
tGSH	PE-MPs	1	75.09	**<0.0001**	27.87
	Temperature	1	113.5	**<0.0001**	42.11
	Exudate	1	2.068	0.1570	0.7677
	PE-MPs x Temperature	1	24.51	**<0.0001**	9.099
	PE-MPs x Exudate	1	2.975	0.0911	1.104
	Temperature x Exudate	1	0.004684	0.9457	0.00179
	PE-MPs x Temperature x Exudate	1	0.06127	0.8056	0.02274
AChE	PE-MPs	1	0.08210	0.7757	0.1537
	Temperature	1	0.04865	0.8264	0.09107
	Exudate	1	0.2332	0.6314	0.4365
	PE-MPs x Temperature	1	0.8336	0.3659	1.560
	PE-MPs x Exudate	1	0.01388	0.9067	0.02597
	Temperature x Exudate	1	2.134	0.1507	3.994
	PE-MPs x Temperature x Exudate	1	3.218	0.0793	6.023
LPO	PE-MPs	1	1.849	0.1803	2.470
	Temperature	1	0.4448	0.5080	0.5942
	Exudate	1	0.7404	0.3938	0.9892
	PE-MPs x Temperature	1	5.385	**0.0246**	7.194
	PE-MPs x Exudate	1	5.365	**0.0249**	7.168
	Temperature x Exudate	1	4.271	**0.0442**	5.706
	PE-MPs x Temperature x Exudate	1	8.795	**0.0047**	11.75
PC	PE-MPs	1	1.953	0.1693	3.205
	Temperature	1	0.4107	0.5250	0.6740
	Exudate	1	2.401	0.1284	3.940
	PE-MPs x Temperature	1	1.299	0.2607	2.131
	PE-MPs x Exudate	1	3.199	0.0806	5.250
	Temperature x Exudate	1	5.860	**0.0197**	9.618
	PE-MPs x Temperature x Exudate	1	2.972	0.0917	4.879
HSP70	PE-MPs	1	14.00	**0.0005**	14.90
	Temperature	1	12.69	**0.0009**	13.51
	Exudate	1	1.463	0.2326	1.558
	PE-MPs x Temperature	1	2.867	0.0972	3.052
	PE-MPs x Exudate	1	4.247	**0.0450**	4.521
	Temperature x Exudate	1	2.821	0.0998	3.003
	PE-MPs x Temperature x Exudate	1	8.925	**0.0045**	9.500

Table S2. Results of a 3-way ANOVA for the biochemical biomarkers responses in the digestive gland of *M. galloprovincialis* with exposure to *A. armata* exudate, PE-MPs and two different temperatures as factors.

Biomarkers	Factor	df	F	*p*	% Variation
CAT	PE-MPs	1	0.8541	0.3600	1.578
	Temperature	1	1.038	0.3133	1.919
	Exudate	1	0.3517	0.5559	0.6498
	PE-MPs x Temperature	1	2.698	0.1070	4.985
	PE-MPs x Exudate	1	0.09021	0.7652	0.1667
	Temperature x Exudate	1	0.7020	0.4063	1.297
	PE-MPs x Temperature x Exudate	1	0.3882	0.5362	0.7173
GST	PE-MPs	1	2.840	0.0984	4.729
	Temperature	1	4.290	**0.0437**	7.142
	Exudate	1	2.471	0.1226	4.114
	PE-MPs x Temperature	1	0.6906	0.4101	1.150
	PE-MPs x Exudate	1	0.3578	0.5526	0.5957
	Temperature x Exudate	1	0.8608	0.3582	1.433
	PE-MPs x Temperature x Exudate	1	0.5527	0.4608	0.9202
tGSH	PE-MPs	1	9.069	**0.0042**	10.30
	Temperature	1	27.05	**<0.0001**	30.73
	Exudate	1	1.992	0.1648	2.262
	PE-MPs x Temperature	1	0.5281	0.4710	0.5999
	PE-MPs x Exudate	1	0.03602	0.8503	0.04091
	Temperature x Exudate	1	2.119	0.1521	2.407
	PE-MPs x Temperature x Exudate	1	0.01939	0.8898	0.02203
AChE	PE-MPs	1	1.498	0.2270	2.339
	Temperature	1	0.00232	0.9618	0.003626
	Exudate	1	0.8623	0.3578	1.346
	PE-MPs x Temperature	1	7.468	**0.0088**	11.66
	PE-MPs x Exudate	1	0.04106	0.8403	0.06411
	Temperature x Exudate	1	0.01092	0.9172	0.01705
	PE-MPs x Temperature x Exudate	1	6.162	**0.0166**	9.622
LPO	PE-MPs	1	0.9362	0.3381	1.687
	Temperature	1	0.2614	0.6115	0.4709
	Exudate	1	0.3402	0.5625	0.6130
	PE-MPs x Temperature	1	0.1158	0.7351	0.2087
	PE-MPs x Exudate	1	0.06438	0.8008	0.1160
	Temperature x Exudate	1	3.595	0.0640	6.478
	PE-MPs x Temperature x Exudate	1	2.183	0.1461	3.933
PC	PE-MPs	1	0.3422	0.5618	0.6752
	Temperature	1	0.1517	0.6989	0.2993
	Exudate	1	0.8619	0.3586	1.701
	PE-MPs x Temperature	1	0.07288	0.7884	0.1375
	PE-MPs x Exudate	1	0.4139	0.5236	0.8167
	Temperature x Exudate	1	0.02356	0.8788	0.04648
	PE-MPs x Temperature x Exudate	1	1.391	0.245	2.745
HSP70	PE-MPs	1	0.5501	0.4621	0.9273
	Temperature	1	6.746	**0.0127**	11.37
	Exudate	1	0.3903	0.5353	0.6579
	PE-MPs x Temperature	1	5.164	**0.0279**	8.706
	PE-MPs x Exudate	1	0.1607	0.6904	0.2710
	Temperature x Exudate	1	0.005231	0.9427	0.008818
	PE-MPs x Temperature x Exudate	1	1.578	0.2156	2.660

Table S3. Results of a 3-way ANOVA for the biochemical biomarkers responses in the muscle of *M. galloprovincialis* with exposure to *A. armata* exudate, PE-MPs and two different temperatures as factors.

Biomarkers	Factor	df	F	*p*	% Variation
AChE	PE-MPs	1	0.001636	0.9679	0.002124
	Temperature	1	12.53	**0.0009**	16.27
	Exudate	1	4.206	**0.0461**	5.462
	PE-MPs x Temperature	1	4.785	**0.0339**	6.213
	PE-MPs x Exudate	1	8.682	**0.0051**	11.27
	Temperature x Exsudate	1	1.699	0.1990	2.207
	PE-MPs x Temperature x Exudate	1	1.664	0.2037	2.161
LPO	PE-MPs	1	0.002159	0.9631	0.004301
	Temperature	1	0.4173	0.5216	0.8311
	Exudate	1	0.6884	0.4111	1.371
	PE-MPs x Temperature	1	0.1528	0.6977	0.3044
	PE-MPs x Exudate	1	0.08021	0.7783	0.1598
	Temperature x Exudate	1	3.377	0.0727	6.727
	PE-MPs x Temperature x Exudate	1	0.5239	0.4729	1.044
PC	PE-MPs	1	2.950	0.0926	3.771
	Temperature	1	24.30	**<0.0001**	31.06
	Exudate	1	0.9438	0.3364	1.207
	PE-MPs x Temperature	1	1.623	0.2090	2.075
	PE-MPs x Exudate	1	0.6620	0.4200	0.8463
	Temperature x Exudate	1	1.731	0.1948	2.213
	PE-MPs x Temperature x Exudate	1	0.2049	0.6529	0.2619

Table S4. Results of a 3-way ANOVA for the energy metabolism biomarkers responses in the muscle of *M. galloprovincialis* with exposure to *A. armata* exudate, PE-MPs and two different temperatures as factors.

Biomarker	Factor	df	F	*p*	% Variation
LDH	PE-MPs	1	0.03904	0.8442	0.06650
	Temperature	1	1.823	0.1833	3.105
	Exudate	1	2.694	0.1072	4.590
	PE-MPs x Temperature	1	1.793	0.1869	3.054
	PE-MPs x Exudate	1	0.4273	0.5164	0.7279
	Temperature x Exudate	1	2.432	0.1255	4.142
	PE-MPs x Temperature x Exudate	1	1.497	0.2272	2.549
Sugar	PE-MPs	1	0.4069	0.5267	0.7412
	Temperature	1	2.648	0.1103	4.825
	Exudate	1	0.1548	0.6958	0.2820
	PE-MPs x Temperature	1	2.490	0.1213	4.535
	PE-MPs x Exudate	1	1.007	0.3208	1.834
	Temperature x Exudate	1	1.042	0.3127	1.897
	PE-MPs x Temperature x Exudate	1	0.007840	0.9298	0.01428
Lipids	PE-MPs	1	0.4014	0.5294	0.6870
	Temperature	1	0.05174	0.8210	0.08855
	Exudate	1	0.8109	0.3724	1.388
	PE-MPs x Temperature	1	6.813	**0.0120**	11.66
	PE-MPs x Exudate	1	0.3077	0.5816	0.5266
	Temperature x Exudate	1	0.1988	0.6577	0.3402
	PE-MPs x Temperature x Exudate	1	1.850	0.1802	3.165
Protein	PE-MPs	1	0.0004154	0.9838	0.0007006
	Temperature	1	9.252	**0.0038**	15.61
	Exudate	1	0.8082	0.3731	1.363
	PE-MPs x Temperature	1	0.04694	0.8294	0.07917
	PE-MPs x Exudate	1	0.9254	0.3409	1.561
	Temperature x Exudate	1	0.1717	0.6804	0.2896
	PE-MPs x Temperature x Exudate	1	0.07607	0.7839	0.1283
ETS	PE-MPs	1	2.809	0.1002	4.993
	Temperature	1	2.114	0.1524	3.758
	Exudate	1	2.789	0.1014	4.957
	PE-MPs x Temperature	1	0.1947	0.6610	0.3460
	PE-MPs x Exudate	1	0.04103	0.8403	0.07292
	Temperature x Exudate	1	0.005964	0.9388	0.01060
	PE-MPs x Temperature x Exudate	1	0.3052	0.5832	0.5425

Table S5. Results of a 3-way ANOVA for byssus production in *M. galloprovincialis* with exposure to *A. armata* exudate, PE-MPs and two different temperatures as factors.

Byssus	Factor	df	F	*p*	% Variation
	PE-MPs	1	0.1403	0.7090	0.1338
	Temperature	1	1.122	0.2928	1.070
	Exudate	1	2.300	0.1334	2.194
	PE-MPs x Temperature	1	0.3109	0.5787	0.2966
	PE-MPs x Exudate	1	0.1540	0.6958	0.1469
	Temperature x Exudate	1	10.65	**0.0016**	10.16
	PE-MPs x Temperature x Exudate	1	11.93	**0.0009**	11.38
